# Exploring the bidirectional causal link between household income status and genetic susceptibility to neurological diseases: findings from a Mendelian randomization study

**DOI:** 10.3389/fpubh.2023.1202747

**Published:** 2023-07-26

**Authors:** Weidong Nong, Gui Mo, Chun Luo

**Affiliations:** Department of Neurology, Affiliated Minzu Hospital of Guangxi Medical University, Nanning, China

**Keywords:** household income status, neurological diseases, causal relationship, instrumental variable, Mendelian randomization study

## Abstract

**Objectives:**

Observational studies have revealed that socioeconomic status is associated with neurological disorders and aging. However, the potential causal effect between the two remains unclear. We therefore aimed to investigate the causal relationship between household income status and genetic susceptibility to neurological diseases using a bidirectional Mendelian randomization (MR) study.

**Methods:**

An MR study was conducted on a large-sample cohort of the European population pulled from a publicly available genome-wide association study dataset, using a random-effects inverse-variance weighting model as the main standard. MR-Egger regression, weighted median, and maximum likelihood estimation were also performed concurrently as supplements. A sensitivity analysis, consisting of a heterogeneity test and horizontal pleiotropy test, was performed using Cochran’s Q, MR-Egger intercept, and MR-PRESSO tests to ensure the reliability of the conclusion.

**Results:**

The results suggested that higher household income tended to lower the risk of genetic susceptibility to Alzheimer’s disease (odds ratio [OR]: 0.740, 95% confidence interval [CI] = 0.559–0.980, *p*-value = 0.036) and ischemic stroke (OR: 0.801, 95% CI = 0.662–0.968, *p*-value = 0.022). By contrast, higher household income tended to increase the risk of genetic susceptibility to Parkinson’s disease (OR: 2.605, 95% CI = 1.413–4.802, *p*-value = 0.002). No associations were evident for intracranial hemorrhage (OR: 1.002, 95% CI = 0.607–1.653, *p*-value = 0.993), cerebral aneurysm (OR: 0.597, 95% CI = 0.243–1.465, *p*-value = 0.260), subarachnoid hemorrhage (OR: 1.474, 95% CI = 0.699–3.110, *p*-value = 0.308), or epilepsy (OR: 1.029, 95% CI = 0.662–1.600, *p*-value = 0.899). The reverse MR study suggested no reverse causal relationship between neurological disorders and household income status. A sensitivity analysis verified the reliability of the results.

**Conclusion:**

Our results revealed that the populations with a superior household income exhibit an increased predisposition of genetic susceptibility to Parkinson’s Disease, while demonstrating a potential decreased genetic susceptibility to ischemic stroke and Alzheimer’s disease.

## 1. Introduction

Neurological diseases can give rise to diverse physical, cognitive, and emotional impairments that can have significant and detrimental impact on the quality of life of an individual. The World Health Organization has reported that neurological conditions account for approximately 6.3% of the global disease burden, making them a primary cause of disability and mortality worldwide (1). With the global population continuing to age, there has been a rise in the prevalence of neurological diseases, particularly those associated with aging, such as Alzheimer’s and Parkinson’s disease (2–4). Over the past few decades, the morbidity rates for certain neurological disorders such as stroke and Alzheimer’s disease have decreased substantially in high-income populations, owing to progress in public health education and awareness of risk factors for these ailments, as well as advancements in medical treatments and interventions (5, 6). By contrast, individuals from low-income populations are at higher risks of developing some types of neurological diseases, such as stroke, due to higher risk factors and lack of access to preventative and specialized stroke care. This can result in a higher risk of disability and a poorer prognosis (7–9). Understanding the link between disease risk and socioeconomic status (SES) holds significance for generating novel hypotheses regarding the influence of environmental and social factors on disease etiology, as well as devising equitable social healthcare policies (10, 11). There is limited evidence regarding the causal connection between household income status and neurological diseases, mainly due to the absence of large-sample cohort studies on the subject. Previous observational studies have reported a relationship between household income and neurological disorders; however observational studies have limitations such as lack of randomization, potential confounding factors, difficulty in controlling variables, and are unable to establish causality due to unaccounted factors that can bias the results (12, 13). Further research is therefore needed to fully understand the nature of this relationship.

Mendelian randomization (MR) is a statistical technique used in epidemiology and genetics to determine the causal relationship between a risk factor and an outcome (14, 15). MR is based on the principles of Mendel’s laws of inheritance, which describe how genetic variants are randomly allocated during meiosis (16). This method uses instrumental variables, specifically genetic variations such as single nucleotide polymorphisms (SNPs) linked to a risk factor of concern (e.g., blood pressure or cholesterol levels), to explore whether the chosen risk factor has an causal impact on the outcome of interest (e.g., heart disease or stroke) (17). In the absence of randomized controlled trials (RCTs), MR studies represent an alternative strategy for causal inference because genetic variants are randomly assigned during meiosis, and therefore add an additional layer of data compared to observational studies. As a result, MR has advantages over traditional observational studies, MR reduces the risk of confounding and reverse causality, making it a superior tool for exploring causality in epidemiological studies (18). Multiple MR studies have effectively employed causal relationship analyses to investigate the links between behavioral exposure, education, socioeconomic conditions, and several diseases (19–22).

This study aimed to utilize an MR approach to establish a bidirectional causal association between genetic susceptibility to common neurological diseases and household income status.

## 2. Materials and methods

### 2.1. Study design and genome-wide association study (GWAS) dataset information

To achieve impartial results, an MR study depends on three fundamental assumptions: (1) the selected genetic instrumental variables (IVs) must be significantly associated with the exposure factor; (2) the IVs should be independent of potential confounders associated with exposure factors and outcomes; and (3) the IVs should affect the outcomes only through the exposure factor (23). This study conducted 14 separate instances of MR analyses designed to explore the bidirectional association between annual household income status and seven neurological diseases.

The study was conducted on data from a large-sample cohort of the European population, pulled from a publicly available GWAS dataset. The variable genetic information involved in this study was extracted from the Integrative Epidemiology Unit GWAS database^1^ (24), which is a publicly available GWAS summary database. Therefore, the requirement for ethical committee approval was waived. The GWAS summary dataset “average total household income before tax” represented the household income status of 397,751 samples originally from the UK biobank database. Annual household income was divided into five intervals: less than 18,000 pounds, 18,000–30,999 pounds, 31,000–51,999 pounds, 52,000–100,000 pounds, and greater than 100,000 pounds. The neurological diseases were represented by Alzheimer’s disease, Parkinson’s disease, ischemic stroke, intracerebral hemorrhage, cerebral aneurysm, subarachnoid hemorrhage, and epilepsy. Detailed information on all the GWAS datasets is listed in Table 1. We followed the sample size and timeliness priority to make the best choices whenever possible. The GWAS datasets of household income and neurological diseases were selected from different consortiums to decrease the potential bias caused by sample overlap. In addition, to minimize racial mismatches, all GWAS datasets involved in this study predominantly included populations of European ancestry.

**Table 1:** Basic information of the genome-wide association study datasets used for the study.

Traits	GWAS ID	Year	Population	Sample size
Exposure factor				Total sample
Household income status (25)	ukb-b-7408	2018	European	397,751
Outcomes				Case/control
Alzheimer’s disease (26)	ieu-b-2	2019	European	21,982/41,944
Parkinson’s disease (27)	ieu-b-7	2019	European	33,674/449,056
Ischemic stroke (28)	ebi-a-GCST006908	2018	European	34,217/406,111
Intracranial hemorrhage (29)	finn-b-I9_INTRACRA	2021	European	2,794/203,068
Cerebral aneurysm (29)	finn-b-I9_ANEURYSM	2021	European	992/203,068
Subarachnoid hemorrhage (29)	finn-b-I9_SAH	2021	European	1,338/201,230
Epilepsy (29)	finn-b-G6_EPLEPSY	2021	European	15,212/29,677

### 2.2. Selection criteria for IVs

The IVs were SNPs, which were filtered according to the three afore-mentioned pivotal assumptions of MR studies. First, the SNPs were matched with a genome-wide statistical significance threshold (*p*-value<5 × 10^−8^). Second, the corresponding linkage disequilibrium was tested to confirm the presence of SNPs in the linkage disequilibrium state, as well as the independence of SNPs, by trimming them within a 0–10,000 kb window at a threshold of *r*^2^<0.001. Third, to evaluate the assumption that the IVs affected the outcomes only through the exposure factor, the potential phenotypes that may have been relevant to the IVs were investigated by searching the human genotype–phenotype association database (PhenoScanner-V_2_) (30). Fourth, SNPs identified as the IVs were further matched to those in the outcome GWAS dataset to establish genetic associations. The summary SNP–phenotype and SNP–outcome statistics were harmonized to ensure effect size alignment, and any palindromic SNPs were excluded. Finally, F-statistics (>10) were used to evaluate the strength of the IVs in order to avoid the influence of weak instrumental bias (31).

### 2.3. MR study and sensitivity analysis

The MR study was performed using a random-effects inverse-variance weighting (IVW) model (32) as the primary standard, as well as three other models [MR-Egger regression (33), weighted median (34), and maximum likelihood (35)] as supplements to evaluate the potential causal relationships between household income status and the seven chosen neurological diseases. The IVW method utilizes a meta-analysis approach to combine Wald estimates for each SNP and obtain an overall estimate of the exposure’s effect on the outcome. In MR-Egger, the IVW estimates are recalculated, removing the constraint of the intercept. The weighted median provides an alternative estimate that remains valid when at least 50% of the instruments are valid. The maximum likelihood model is similar to IVW, assuming the absence of heterogeneity and horizontal pleiotropy. Under the fulfillment of these assumptions, the results will be unbiased, with smaller standard errors compared to IVW. The reverse MR study evaluated the potential causal relationship between the seven neurological diseases and household income status using the same methods. In addition, a sensitivity analysis was performed to measure the reliability and stability of the conclusion. The sensitivity analysis consisted of (1) a Cochran’s Q test (according to the IVW model or MR-Egger regression model); (2) a horizontal pleiotropy test using an MR-Egger intercept (36) and an MR-PRESSO test (37); and (3) a “leave-one-out” test (each SNP was dropped successively and the IVW analysis was repeated to identify whether any specific SNP drove the causal relationship estimate). The results are reported as odds ratios (ORs) with corresponding 95% confidence intervals (CIs) and *p*-values, as well as scatterplots. The evidential threshold for the MR analysis was defined as *p*-value <0.004 (0.05/14), according to the Bonferroni correction method. A *p*-value <0.05 but above the Bonferroni corrected evidential threshold was regarded as a potential association. A *p*-value <0.05 was also considered significant in the sensitivity analysis. R v4.0.3 software, equipped with the “TwoSampleMR” (38) and “MR-PRESSO” (37) packages, was used to process and visualize the study.

## 3. Results

### 3.1. MR study

The sample overlap of the seven GWAS datasets and the UK-biobank database were as follows: Alzheimer’s disease: 0%; Parkinson’s disease: none available; ischemic stroke: 0%; intracranial hemorrhage: 0%; cerebral aneurysm: 0%; subarachnoid hemorrhage: 0%; and epilepsy: 0%. Sample overlap rates between household income and neurological diseases were therefore shown to be extremely low.

The numbers of SNPs that were ultimately identified as the IVs in the different outcome datasets were 42 (Alzheimer’s disease, intracranial hemorrhage, cerebral aneurysm, subarachnoid hemorrhage, and epilepsy) and 44 (Parkinson’s disease, ischemic stroke), respectively. The F-statistic scores of all these selected SNPs were over 10 (Alzheimer’s disease: 57.64, intracranial hemorrhage: 57.87, cerebral aneurysm: 57.77, subarachnoid hemorrhage: 57.77, epilepsy: 57.77, Parkinson’s disease: 57.49, and ischemic stroke: 57.76), indicating a low risk of weak-instrument bias.

**Figure 1:**
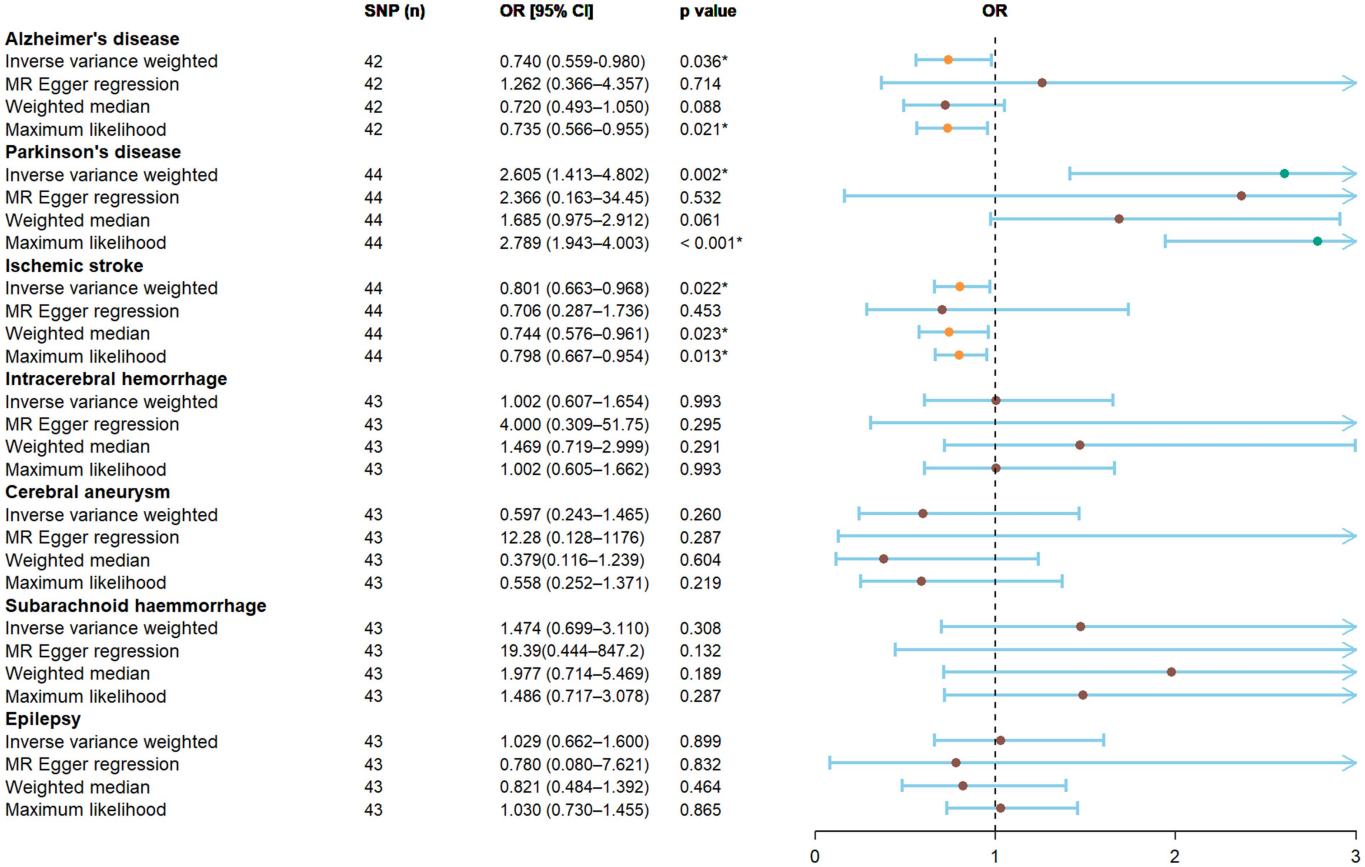
The result of the MR study illustrated by forest plot. The causal relationship between household income status and neurological diseases was evaluated using an MR study. OR, odds ratio; CI, confidence interval; MR, Mendelian randomization; SNP, single nucleotide polymorphism.

According to the random-effects IVW model results, higher household income, as the primary standard, tended to lower the risk of genetic susceptibility to Alzheimer’s disease (OR: 0.740, 95% CI = 0.559–0.980, *p*-value = 0.036) and ischemic stroke (OR: 0.801, 95% CI = 0.662–0.968, *p*-value = 0.022). By contrast, higher household income tended to increase the risk of genetic susceptibility to Parkinson’s disease (OR: 2.605, 95% CI = 1.413–4.802, *p*-value = 0.002). However, no evidence was found of a potential causal relationship between household income status and intracranial hemorrhage (OR: 1.002, 95% CI = 0.607–1.653, *p*-value = 0.993), cerebral aneurysm (OR: 0.597, 95% CI = 0.243–1.465, *p*-value = 0.260), subarachnoid hemorrhage (OR: 1.474, 95% CI = 0.699–3.110, *p*-value = 0.308), or epilepsy (OR: 1.029, 95% CI = 0.662–1.600, *p*-value = 0.899). The results of our weighted median and maximum likelihood estimation models supported these conclusions. The MR-Egger regression model results, however, did not show significant differences. In summary, according to the Bonferroni correction standard, this MR study revealed that the population with a higher household income tended to have a greater risk of genetic susceptibility to Parkinson’s disease. The results also suggest a potentially negative relationship between Alzheimer’s disease and ischemic stroke. Detailed information is displayed in the forest plot in Figure 1, and is illustrated as a scatterplot in Supplementary Figure 1.

### 3.2. Sensitivity analyses

The results of our Cochran’s Q test indicated certain heterogeneity among the IVs in terms of Parkinson’s disease and epilepsy (Table 2). The random effects IVW model was therefore used to minimize the effect of heterogeneity in the MR study. No horizontal pleiotropy was detected using the MR-Egger intercept and MR-PRESSO tests (Table 2). In addition, the “leave-one-out” method indicated that no specific SNP among the IVs significantly affected the overall result (Supplementary Figure 2). In general, the sensitivity analysis verified the robustness of the conclusions.

**Table 2:** Results of heterogeneity and horizontal pleiotropy tests.

Diseases	Heterogeneity test		Horizontal pleiotropy test	
	MR-Egger regression	IVW model	MR-Egger intercept	MR-PRESSO test
Alzheimer’s disease	0.189	0.195	0.391	0.153
Parkinson’s disease	<0.001	<0.001	0.943	0.163
Ischemic stroke	0.195	0.223	0.780	0.202
Intracranial hemorrhage	0.793	0.784	0.286	0.805
Cerebral aneurysm	0.254	0.225	0.193	0.296
Subarachnoid hemorrhage	0.375	0.339	0.180	0.382
Epilepsy	0.002	0.003	0.809	–

### 3.3. Reverse MR study and sensitivity analyses

The numbers of SNPs that were ultimately identified as the IVs for different neurological diseases in the reverse MR study were 18 (Alzheimer’s disease), 22 (Parkinson’s disease), 7 (ischemic stroke), 1 (intracranial hemorrhage) and 0 (cerebral aneurysm/subarachnoid hemorrhage/epilepsy).

Based on the random-effects IVW model results, the reverse MR study suggested no reverse causal relationships between the neurological diseases and household income status. More detailed information on this analysis is displayed in Table 3.

**Table 3:** Results of the reverse MR study.

Disease	SNPs (n)	OR (95%CI)	*p*-value
**Alzheimer’s disease**			
Inverse variance weighted	18	1.003 (0.993–1.012)	0.567
MR Egger regression	18	1.003 (0.990–1.017)	0.654
Weighted median	18	1.003 (0.993–1.013)	0.551
Maximum likelihood	18	1.003 (0.996–1.010)	0.443
**Parkinson’s disease**			
Inverse variance weighted	22	1.019 (1.001–1.036)	0.036
MR Egger regression	22	1.015 (0.982–1.048)	0.120
Weighted median	22	1.014 (0.999–1.027)	0.054
Maximum likelihood	22	1.019 (1.010–1.028)	<0.001*
**Ischemic stroke**			
Inverse variance weighted	7	0.978 (0.944–1.014)	0.241
MR Egger regression	7	0.916 (0.685–1.223)	0.578
Weighted median	7	0.982 (0.943–1.022)	0.375
Maximum likelihood	7	0.978 (0.948–1.009)	0.162
Intracerebral hemorrhage			
Inverse variance weighted	1	NA	NA
MR Egger regression	1	NA	NA
Weighted median	1	NA	NA
Maximum likelihood	1	NA	NA
**Cerebral aneurysm**			
Inverse variance weighted	0	NA	NA
MR Egger regression	0	NA	NA
Weighted median	0	NA	NA
Maximum likelihood	0	NA	NA
**Subarachnoid hemorrhage**			
Inverse variance weighted	0	NA	NA
MR Egger regression	0	NA	NA
Weighted median	0	NA	NA
Maximum likelihood	0	NA	NA
**Epilepsy**			
Inverse variance weighted	0	NA	NA
MR Egger regression	0	NA	NA
Weighted median	0	NA	NA
Maximum likelihood	0	NA	NA

OR, odds ratio; CI, confidence interval; MR, Mendelian randomization; SNPs, single nucleotide polymorphisms; NA, Not available. *statistically significant difference with a *p*-value less than 0.05 (*p* < 0.05).

## 4. Discussion

Epidemiological research has extensively investigated the impact of SES on neurological diseases (39). Household income, as a crucial component of SES, has consistently been associated with the probability of developing neurological diseases (40, 41). However, a comprehensive investigation of the causal relationship between household income and neurological diseases is still necessary. This study aimed to address this research gap by conducting a bidirectional two-sample MR analysis to examine the causal relationship between household income and neurological diseases. To the best of our knowledge, this is the first study to investigate the genetic risk aspect. According to our results, individuals belonging to households with higher income tended to have reduced genetic risks of ischemic stroke and Alzheimer’s disease. In contrast, household income exhibited a potentially positive correlation with Parkinson’s Disease. We found no significant association between household income and the risk of developing epilepsy, intracranial hematoma, cerebral aneurysm, or subarachnoid hemorrhage.

Similarly, compelling evidence has previously suggested a correlation between low household income and the incidence of ischemic stroke, with higher household income levels observed as the incidence of stroke decreased (5, 42). The results of that study indicated that some of the known classical risk factors for stroke were overrepresented in groups with low SES (43). The heightened risk of stroke in low-income groups may be partly attributed to lifestyle factors, specifically smoking, high alcohol consumption, and obesity (40, 44). The prevalence of diabetes is also considerably higher in this group, which may contribute to the increased risk (45). After factoring in these conventional risk factors, the link of elevated risk of stroke with SES was mitigated, yet the incidence of stroke in this group remained higher (46). Evidence has also suggested that individuals with lower incomes have more limited access to healthcare and preventative resources compared to those with higher incomes (47). This lack of access, coupled with neglect of essential health maintenance behaviors such as annual medical checkups and adherence to secondary prevention medications, may further exacerbate the risk of stroke. Lower household incomes is potentially associated with an increased risk of ischemic stroke, which can be attributed to a range of underlying molecular biological mechanisms. Chronic inflammation resulting from higher levels of chronic stress and limited healthcare access promotes atherosclerosis, leading to plaque formation and arterial narrowing (48–51). Limited management of cardiovascular diseases, such as hypertension, due to inadequate resources contributes to vascular dysfunction through impaired regulation of blood vessel tone, endothelial dysfunction, and increased oxidative stress (52). Additionally, epigenetic influences influenced by socio-economic factors may impact the expression and function of genes related to inflammation, vascular function, and coagulation (53).

According to our findings, which are similar to those of several previous studies, low-income status was associated with an increased risk of Alzheimer’s disease (54, 55). However, previous research has been limited by selection bias and the heterogeneity of comparison groups. Compared to previous MR studies on the relationship between income and incidence of Alzheimer’s disease, our study utilized a bidirectional MR method to analyze the relationship between household income status and genetic susceptibility to Alzheimer’s disease, which is an improvement over previous unidirectional MR studies that only examined the impact of household income on Alzheimer’s disease (55). Additionally, the exposure dataset (household income) and the seven disease datasets used in this study were obtained from different databases with a low sample overlap rate, increasing the reliability of our conclusions. Low-income individuals may have higher risks of developing Alzheimer’s disease due to various factors such as limited access to healthcare (resulting in untreated chronic conditions), lower levels of education (leading to less cognitive reserve), unhealthy lifestyle behaviors, and higher levels of chronic stress, all of which can cause inflammation and damage to brain cells (54, 56, 57). In the previous study that explored the connection between genetic factors and household income, researchers identified four genome-wide significant SNPs that exhibited a significant association with income levels (58). These SNPs resulted in the discovery of two distinct genomic regions, wherein genes previously implicated in intellectual disabilities, synaptic plasticity, and schizophrenia were found, indicating potential shared genetic mechanisms underlying income disparities and Alzheimer’s disease. This study suggests that individuals with lower household incomes may be more susceptible to Alzheimer’s disease. The utilization of a bidirectional MR method and data from different databases increased the reliability of our findings.

The association between SES and Parkinson’s disease has not been studied extensively on a global level, and the existing findings on the subject have been inconclusive. In a Canadian population-based study that used census data, SES categories were determined by the average household income, and the results indicated an inverse relationship between SES and the incidence of Parkinson’s disease (59). Specifically, the incidence and prevalence of Parkinson’s disease were significantly higher in the lower quintile of urban areas (59). Another population-based study in Sweden explored the relationship between SES and risk of Parkinson’s disease, with SES determined by a surrogate measure such as occupation (60). This study found that lower SES was associated with a lower incidence of Parkinson’s disease, which is consistent with the findings of our study. Individuals in low-income households may have a reduced risk of Parkinson’s disease, due to household income-related factors such as smoking and physical activity, which are strongly associated with a lower risk (60). Higher levels of physical activity and smoking are more common in low-income groups, especially those with manual labor occupations (61). The biological functions of SNPs as IVs and their impact on Parkinson’s disease warrant further investigation. Hill et al. identified 30 independent loci associated with individual income, which may be implicated in the biological processes underlying gamma-aminobutyric acid (GABA)ergic and serotonergic neurotransmission. GABA and serotonin are neurotransmitters that play critical roles in regulating brain function and behavior (62). While Parkinson’s disease is primarily characterized by the degeneration of dopamine-producing cells in the substantia nigra, emerging evidence suggests additional alterations in neurotransmitter systems, including the GABAergic and serotonergic pathways (63, 64). Among the 30 genetic loci reported by Hill et al., we identified six SNPs (rs11588857, rs6699397, rs32940, rs10429582, rs2332719, rs784256) that are shared with the selected IVs in our study. This finding suggests that the SES indicated by household income may be associated with the likelihood of progression of Parkinson’s disease through biological mechanisms associated with GABAergic and serotonergic pathways. Nevertheless, additional direct evidence is necessary to substantiate this hypothesis.

The bidirectional MR study design carries a significant advantage in terms of effectively avoiding the influence of reverse causes and reducing residual confounding. However, certain limitations of this study need to be acknowledged as well. First, our heterogeneity test results revealed some heterogeneity among the IVs in terms of Parkinson’s disease and epilepsy. Although the random effects IVW model was used to minimize the effect of heterogeneity in the MR study as much as possible, this heterogeneity should not be overlooked. Second, various MR study assumptions have distinct advantages and disadvantages, which may lead to inconsistent or contradictory results. Therefore, the results of our study need to be interpreted with some caution. Third, the GWAS dataset we used primarily drew from populations of European descent to evade confounding due to population stratification. As a result, the current findings may not be generalizable to other ethnic groups, and additional research is necessary to comprehend how these outcomes may apply to diverse populations.

## 5. Conclusion

This study explored the causal relationship between household income status and neurological diseases using a bidirectional MR study based on datasets with millions of individual samples. Our results revealed that the populations with a superior household income exhibit an increased predisposition of genetic susceptibility to Parkinson’s Disease, while demonstrating a potential decreased genetic susceptibility to ischemic stroke and Alzheimer’s disease.

## Data availability statement

The datasets presented in this study can be found in online repositories. The names of the repository/repositories and accession number(s) can be found in the article/Supplementary material.

## Author contributions

WN was responsible for conception and article writing. GM was responsible for data mining. CL was responsible for scientific supervision. All authors reviewed and approved the final manuscript.

## Funding

This research received no specific grant from any funding agency in the public, commercial, or not-for-profit sectors.

## Conflict of interest

The authors declare that the research was conducted in the absence of any commercial or financial relationships that could be construed as a potential conflict of interest.

## Publisher’s note

All claims expressed in this article are solely those of the authors and do not necessarily represent those of their affiliated organizations, or those of the publisher, the editors and the reviewers. Any product that may be evaluated in this article, or claim that may be made by its manufacturer, is not guaranteed or endorsed by the publisher.

## Footnotes

^1^ https://gwas.mrcieu.ac.uk/

## Abbreviations

MR: Mendelian randomization
SNP: single nucleotide polymorphism
GWAS: genome-wide association study
IV: instrumental variable
IVW: inverse-variance weighting
OR: odds ratio
CI: confidence interval
SAH: Subarachnoid hemorrhage
GABA: gamma-aminobutyric acid
