# Supplementary figures and images for: Exploring the bidirectional causal link between household income status and genetic susceptibility to neurological diseases: findings from a Mendelian randomization study
Source: Front Public Health. 2023 Jul 26;11:1202747. doi: 10.3389/fpubh.2023.1202747 (PMC10411908; doi:10.3389/fpubh.2023.1202747)

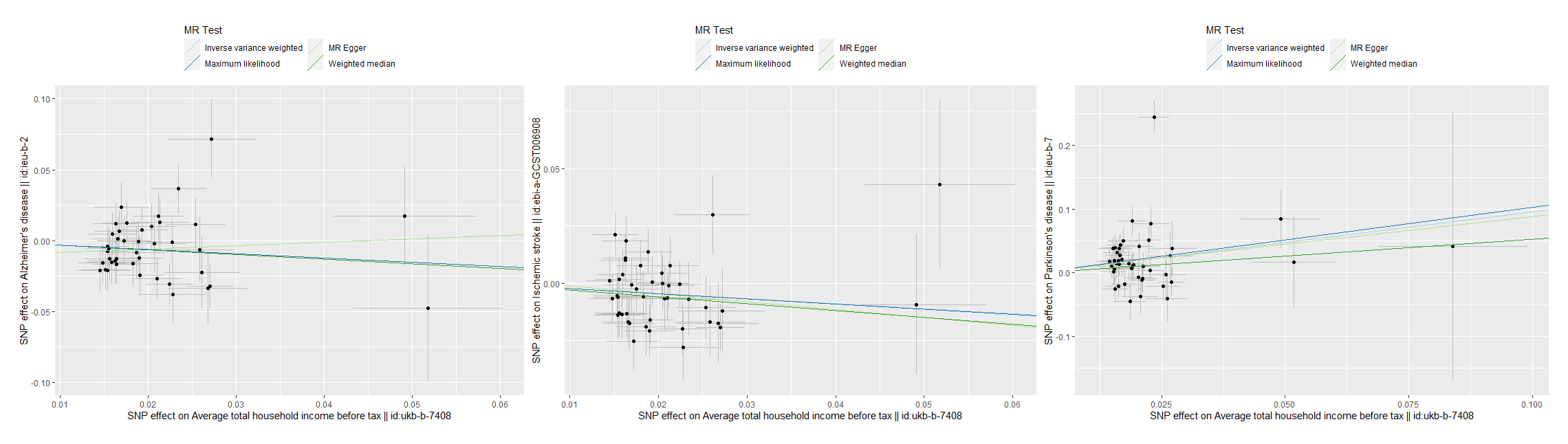

Supplement: Supplementary file 1 [file Image_1.PNG]

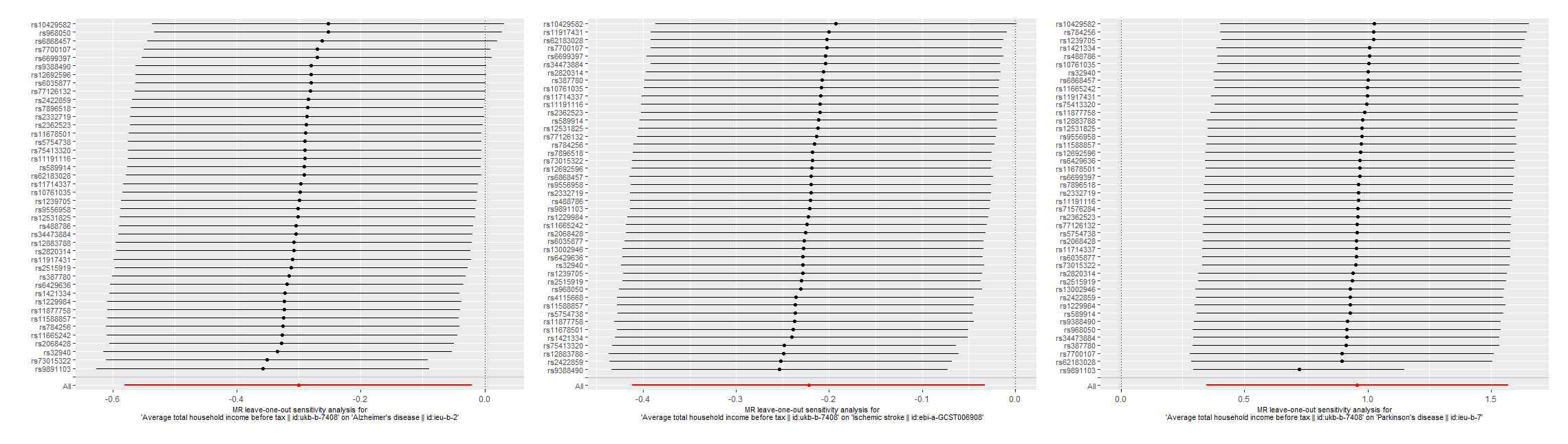

Supplement: Supplementary file 2 [file Image_2.PNG]
